# Aberrant Expression of β-Catenin Correlates with Infiltrating Immune Cells and Prognosis in NSCLC

**DOI:** 10.3389/pore.2021.1609981

**Published:** 2021-10-26

**Authors:** Hongmei Zheng, Yue Ning, Yang Yang, Yuting Zhan, Haihua Wang, Qiuyuan Wen, Jinwu Peng, Songqing Fan

**Affiliations:** ^1^ Department of Pathology, The Second Xiangya Hospital, Central South University, Changsha, China; ^2^ Department of Pathology, Xiangya Basic Medical School, Central South University, Changsha, China

**Keywords:** biomarker, NSCLC, β-catenin, tumor microenvironment, tumor immunity

## Abstract

**Aims:** β-catenin is a critical regulating factor of the Wnt pathway, which is closely linked to tumorigenesis, tumor growth, metastasis, and tumor immunity. Our study focused on exploring the relationship between β-catenin and clinicopathological features, prognosis, as well as infiltrating immune cells and immune scores, so as to illustrate its clinical significance in NSCLC.

**Materials and Methods:** The β-catenin mRNA (*CTNNB1*) and protein expression data were downloaded from the UALCAN and the UCSC Xena website, respectively. All tumor-immune infiltrating cells’ data were downloaded from the TIMER platform and immune scores were downloaded from ESTIMATE website. The expression of β-catenin protein in our cohort was measured by immunohistochemistry.

**Results:** β-catenin mRNA level was higher in lung adenocarcinoma (LUAD) compared to normal tissues (*p* < 0.001) and was related to overall survival (OS) (*p* < 0.001) and post-progression survival (PPS) (both *p =* 0.049) in LUAD. Aberrant β-catenin protein expression was higher in male and lung squamous cell carcinoma (LUSC) patients (both *p* = 0.001). Also, it was considered to be a prognosis factor independently (*p* = 0.034). In addition, β-catenin protein was negatively correlated with CD8^+^T cells (r = −0.128, *p* = 0.008), neutrophils (r = −0.198, *p* < 0.001), immune score (r = −0.109, *p* = 0.024), stromal score (r = −0.097, *p* = 0.045), and ESTIMATE score (r = −0.113, *p* = 0.020).

**Conclusions:** Aberrant β-catenin protein expression was evidently higher in NSCLC and might serve as a biomarker for poor prognosis. Most importantly, β-catenin protein might play an important part in tumor immunity and the tumor microenvironment by inhibiting the infiltration of CD8+ T cells and neutrophils.

## Introduction

Lung cancer, which is one of the most principal malignant cancers with increasing incidence rates year by year, often occurs in bronchial mucosal epithelium and alveoli [1,2]. It is divided into SCLC (small-cell lung cancer) and NSCLC (non-small cell lung cancer), and then further divided into squamous cell carcinoma, adenocarcinoma, and large-cell lung cancer [3]. Most patients are asymptomatic, and the tumor may progress when the patient has respiratory tract or tumor-related symptoms [4]. Although chemoradiotherapy and targeted therapy (such as EGFR-TKI, epidermal growth factor receptor-tyrosine kinase inhibitor) have brought unprecedented survival benefits to some patients, all these treatments are prone to drug resistance and have some side effects [5,6]. In recent years, immuno-checkpoint inhibitor (ICI), represented by monoclonal antibodies against programmed death-1 (PD-1)/programmed death ligand-1 (PD-L1), can cause long-term remission and even cure 20–30% of patients. It is a further sign of revolutionary progress in the field of lung cancer treatment, but not all patients can benefit from immunotherapy, and the overall effective rate is low [7,8]. The occurrence and development of NSCLC is an extremely complex process involving multiple steps, multiple factors, and multiple genes. Consequently, it is urgent to study the exact pathogenesis of non-small cell lung cancer and find accurate biomarkers for predicting prognosis and searching for new clues for therapeutic targets.

β-catenin is a multifunctional protein, which was first discovered as a mammalian cell adhesion complex in 1990. It can interact with cadherin, participate in cell adhesion, and form cytoskeletons [9,10]. The imbalance of β-catenin structure and signal properties often leads to disease and uncontrolled growth of cancer [11]. Also, it is a critical regulating factor of the Wnt pathway. When the classical Wnt pathway is activated, the cytoplasmic β-catenin is transferred to the nucleus and then interacts with some transcription factors (such as LEF, TCF, and BCL-9) to further activate target genes of the pathway [12,13]. Abnormal activation of the Wnt/β-catenin pathway exists widely in different types of malignant tumors, such as lung cancer, pancreatic cancer, liver cancer, and colorectal cancer, and is closely associated with tumorigenesis, tumor growth, metastasis, and tumor immunity [14–16].

Tumor microenvironment, as the living environment of tumor cells, is composed of various cytokines/chemokines, immune cells, tumor-related fibroblasts, inflammatory cells, and microvessels and has a significant impact on the treatment response and overall prognosis of tumor patients [17,18]. Reshaping the immune response of the immune microenvironment is helpful to improve the prognosis of patients. Crosstalk between Wnt/β-catenin signaling and immune cells has both positive and negative effects on tumor progression. It helps to maintain and renew the leucocytes, but also promotes immune tolerance and limits anti-tumor response [19]. The relevance between Wnt/β-catenin signaling and immune microenvironment is complicated in NSCLC. Therefore, this study will explore the potential correlation between β-catenin protein and clinicopathological/prognostic features, as well as its association with tumor-infiltrating immune cells and immune score.

## Materials and Methods

### Data Collection

The expression of *CTNNB1* (β-catenin mRNA) and β-catenin protein in The Cancer Genome Atlas (TCGA) database was downloaded from the UALCAN (http://ualcan.path.uab.edu/) and the UCSC Xena website (http://xena.ucsc.edu/), respectively. All tumor-immune infiltrating cells data (including macrophages, CD4^+^T-cells, B-cells, neutrophils CD8^+^T-cells, and dendritic cells) were obtained from the Tumor Immune Estimation Resource (TIMER) platform (https://cistrome.shinyapps.io/timer/). The immune score, stromal score, and estimate score were downloaded from ESTIMATE (https://bioinformatics.mdanderson.org/estimate/). K-M plotter (http://kmplot.com/analysis/index.php?p=service#) was applied to analyze the potential prognostic value of *CTNNB1* in NSCLC, which was divided into low and high groups according to the median level [20].

### Patients Cohorts

We collected 273 cases of NSCLC postoperative samples from Central South University, The Second Xiangya Hospital. There were 133 cases of LUSC, 140 of LUAD, and 106 non-cancerous lung tissues from 2003 to 2013, including 70 female and 203 male patients with a median age of 56 years. Of the cases, 132 were at clinical stage I and II and 141 cases were at stage III. 154 cases had lymph node metastasis and 119 had no lymph node metastasis. At the last follow-up, 164 patients had survived and 109 patients had died. All patients had complete follow-up and clinical data. None of them had received treatment before surgery. Each case had definite clinical stage and pathological diagnosis on the basis of the Eighth Edition Lung Cancer Stage Classification and the WHO histological classification, respectively [21].

### IHC and Scores

The β-catenin protein with a dilution of 1:100 (Rabbit mAb #8480, Cell Signaling Technology) was examined according to the protocols as described before [22]. In short, the slides were dewaxed and rehydrated, repaired at high temperature with sodium citrate for 10 min, blocked with 3% hydrogen peroxide, and finally incubated with primary antibody in a wet box at 4°C overnight. Each experiment included negative and positive controls.

The Immunohistochemical score was independently performed under 200x light microscopy by YZ and SF who were blinded to patients’ clinical information. The score evaluation was as follows [23]: normal expression was defined as more than 70% of the cell membrane having positive staining for β-catenin and less than 60% of the cytoplasm or the nucleus showing positive staining, the others were defined as aberrant expression. All discordances were resolved through discussion.

### Statistical Analysis

SPSS 24.0 was applied to all statistical analyses. The correlation between β-catenin protein level and clinicopathological factors of NSCLC was examined by chi-square test. Besides, the relationship among β-catenin protein, tumor-immune infiltrating cells, and immune score was examined by Spearman’s correlation test. Survival situation was tested by Kaplan-Meier analysis and Log-rank test. Cox hazards model was applied to evaluate the independent prognostic factors of NSCLC. *p <* 0.05 (two-sided) was considered to be statistically significant.

## Results

### Correlation Between Aberrant Expression of β-catenin and Clinicopathological Variables in NSCLC

**FIGURE 1:**
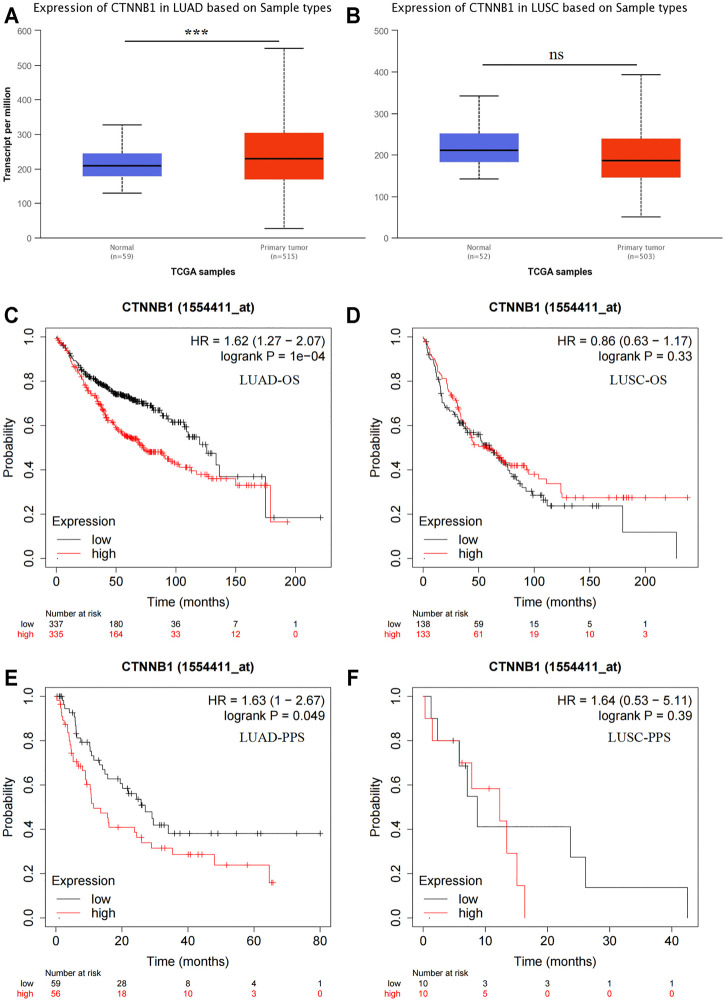
Bioinformatics analysis of β-catenin mRNA expression (*CTNNB1*). Expression of *CTNNB1* in LUAD **(A)** and LUSC **(B)**. OS analysis of *CTNNB1* in LUAD **(C)** and LUSC **(D)**. PPS analysis of *CTNNB1* in LUAD **(E)** and LUSC **(F)**. OS, overall survival; PPS, post-progression survival.

In UALCAN database, *CTNNB1* mRNA expression was up-regulated in LUAD (Figure 1, *p* < 0.001) but not in LUSC (*p* > 0.05). We also detected the prognostic value of *CTNNB1*. Low *CTNNB1* mRNA expression was related to better OS (overall survival) and PPS (post progression survival) in LUAD (Figure 1, both *p* < 0.05), but had no correlation in LUSC (both *p* > 0.05).

**FIGURE 2:**
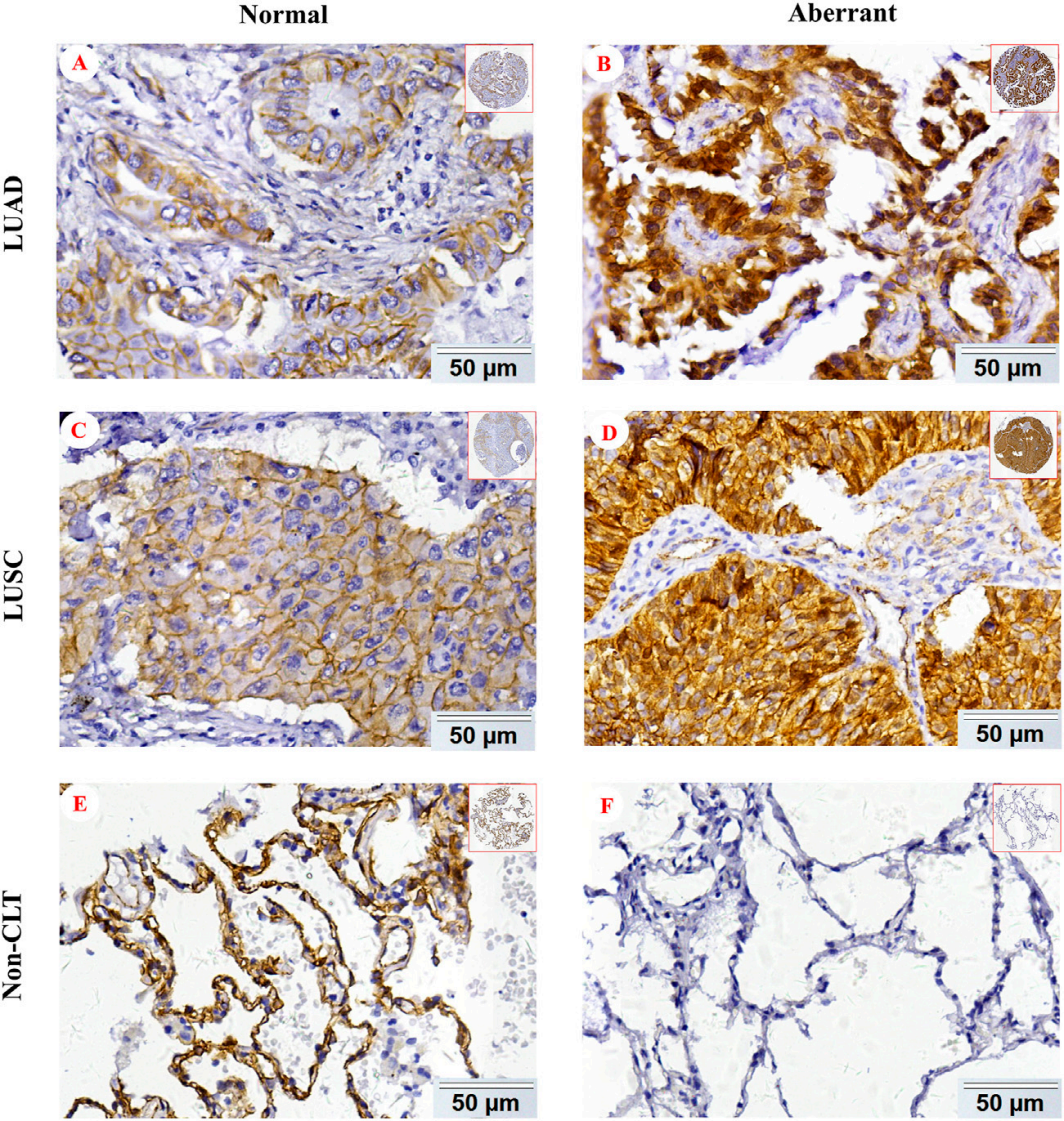
Representative IHC images of β-catenin protein in NSCLC. Normal β-catenin protein expression in LUAD **(A)**, LUSC **(C)**, and Non-CLT **(E)**. Aberrant β-catenin protein expression in LUAD **(B)**, LUSC **(D)**, and Non-CLT **(F)**. Non-CLT: non-cancerous control lung tissue.

**FIGURE 3:**
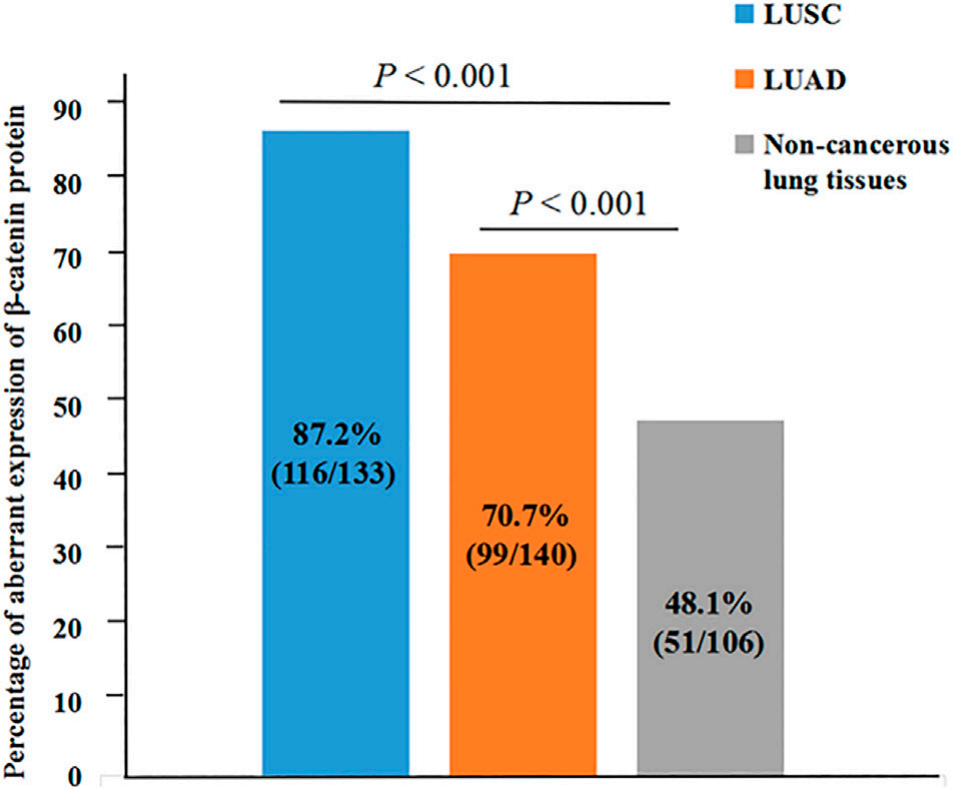
Aberrant expression rate of β-catenin protein in NSCLC and non-cancerous lung tissues. Aberrant β-catenin protein expression rate was increased in LUSC and LUAD (both *p* < 0.001).

In order to explore β-catenin protein expression in our patient cohort, we used IHC to detect the subcellular localization and expression of β-catenin protein in NSCLC and Non-CLT (non-cancerous control lung tissue). β-catenin protein was chiefly distributed in the cell membrane/cytoplasm, as well as in the nucleus (Figure 2). Aberrant β-catenin protein expression was 78.8% (215/273) in NSCLC but was 48.1% (51/106) in non-cancerous control lung tissues (Figure 3). Specifically, β-catenin protein aberrant expression was 70.7% (99/140) in LUAD and 87.2% (116/133) in LUSC tissues. We further analyzed the link between β-catenin protein and clinicopathological variables (including pathological grade, clinical stage, age, gender, and histological type) by chi-square test. Results in Table 1 showed that aberrant β-catenin protein expression rate was increased in male and LUSC patients (both *p* = 0.001) compared to female and LUAD patients. It is worth noting that aberrant β-catenin protein expression rate was higher in clinical stage III patients compared to stage I patients, but the statistical difference was weak (*p* = 0.078). No obvious statistical difference was found in other clinicopathological variables (*p >* 0.05).

**TABLE 1:** Association between aberrant expression of β-catenin protein and clinicopathological features of NSCLC (*n* = 273).

Variables	β-catenin		
	Normal (%)	Aberrant (%)	*p*
Age (years)			
<60 (*n* = 177)	37 (20.9%)	140 (79.1%)	0.851
≥60 (*n* = 96)	21 (21.9%)	75 (78.1%)	
Gender			
Female (*n* = 70)	25 (35.7%)	45 (64.3%)	0.001*
Male (*n* = 203)	33 (16.3%)	170 (83.7%)	
Histological type			
LUAD (*n* = 140)	41 (29.3%)	99 (70.7%)	0.001*
LUSC (*n* = 133)	17 (12.8%)	116 (87.2%)	
Pathological grade			
Well/moderate (*n* = 128)	31 (24.2%)	97 (75.8%)	0.259
Poor (*n* = 145)	27 (18.6%)	118 (81.4%)	
Clinical stage			
Stage I and II (*n* = 132)	34 (25.8%)	98 (74.2%)	0.078
Stage III (*n* = 141)	24 (17.0%)	117 (83.0%)	
LNM status			
LNM (*n* = 154)	29 (18.8%)	125 (81.2%)	0.267
No LNM (*n* = 119)	29 (24.4%)	90 (75.6%)	

*p < 0.05.

LUAD, lung adenocarcinoma; LNM, lymph node metastasis; LUSC, lung squamous cell carcinoma.

### Effects of Aberrant β-Catenin Protein Expression on Prognosis of NSCLC Patients

**FIGURE 4:**
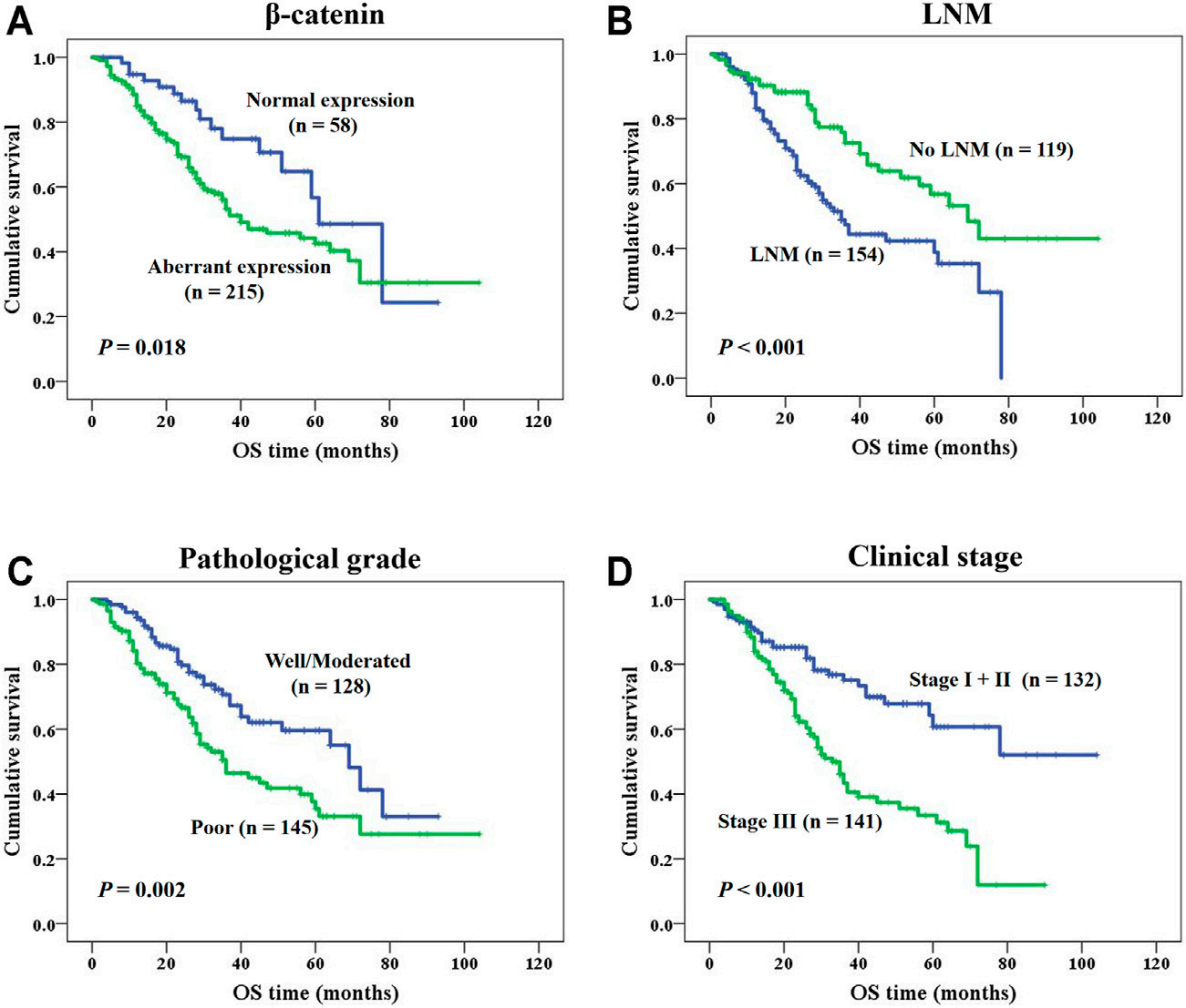
K-M survival analysis of NSCLC patients. **(A)** NSCLC patients with aberrant β-catenin protein expression had a shorter OS (*p* = 0.018). **(B)** NSCLC patients with LNM had a shorter OS (*p* < 0.001). **(C)** Patients with poor differentiated NSCLC had a shorter OS (*p =* 0.002). **(D)** NSCLC patients with clinical stage III had a shorter OS (*p <* 0.001).

In this study, survival status of NSCLC patients with different clinicopathological variables was analyzed through Kaplan-Meier survival analysis. For patients with NSCLC, the overall survival of patients with aberrant β-catenin protein expression was remarkably shorter than that of patients with normal protein expression (*p* = 0.018, Figure 4A). In addition, shorter survival time was identified in cases with LNM (lymph node metastasis; *p* < 0.001, Figure 4B), poor differentiation (*p* = 0.002, Figure 4C), and clinical stage III (*p* < 0.001, Figure 4D). We also analyzed the prognostic value of β-catenin protein in LUAD and LUSC independently (Supplementary Figure S1). We found that β-catenin protein had prognostic value in LUAD, but not in LUSC.

Moreover, we also studied whether β-catenin protein expression status could be utilized as a prognostic factor for NSCLC patients independently. As shown in Table 2, we identified aberrant expression of β-catenin protein as a poor prognostic factor independently (*p* = 0.034), along with clinical stage III (*p* = 0.003), poor differentiation (*p* = 0.015), and with LNM (*p* = 0.019).

**TABLE 2:** Univariate and multivariate analysis for OS in NSCLC patients.

Parameters	Univariate analysis			Multivariate analysis		
	Average survival time (SE)	95% CI	*p*	Exp (B)	95.0% CI	*p*
β-catenin						
Aberrant expression	53.981 (3.506)	(47.110, 60.852)	0.018*	0.552	(0.319, 0.956)	0.034*
Normal expression	61.862 (5.423)	(51.233, 72.491)				
Clinical stage						
Stage I and II	72.615 (4.644)	(63.513, 81.717)	0.000*	0.521	(0.336, 0.806)	0.003*
Stage III	41.670 (2.947)	(35.894, 47.447)				
LNM status						
LNM	43.467 (2.698)	(38.178, 48.755)	0.000*	1.681	(1.089, 2.596)	0.019*
No LNM	67.481 (4.643)	(58.382, 76.581)				
Pathological grade						
Well and moderate	60.332 (3.826)	(52.833, 67.831)	0.002*	0.614	(0.414, 0.910)	0.015*
Poor	50.220 (4.116)	(42.153, 58.288)				
Histological type						
LUAD	50.983 (3.365)	(44.343, 57.534)	0.798	1.382	(0.919, 2.078)	0.120
LUSC	61.293 (4.685)	(52.109, 70.477)				
Gender						
Female	60.240 (4.789)	(50.854, 69.625)	0.077	0.672	(0.417, 1.085)	0.104
Male	53.347 (3.748)	(46.001, 60.692)				
Age						
<60	58.663 (4.008)	(50.806, 66.519)	0.107	0.816	(0.552, 1.206)	0.307
≥60	48.535 (4.352)	(40.006, 57.064)				

*p < 0.05.

OS, overall survival; CI, confidence interval; SE, standard error; Exp(β), odds ratio; LUAD, lung adenocarcinoma; LNM, lymph node metastasis; LUSC, lung squamous cell carcinoma.

### Correlation Analysis Between β-Catenin Protein Expression and Immune Cell Infiltration Level and Immune Score

**FIGURE 5:**
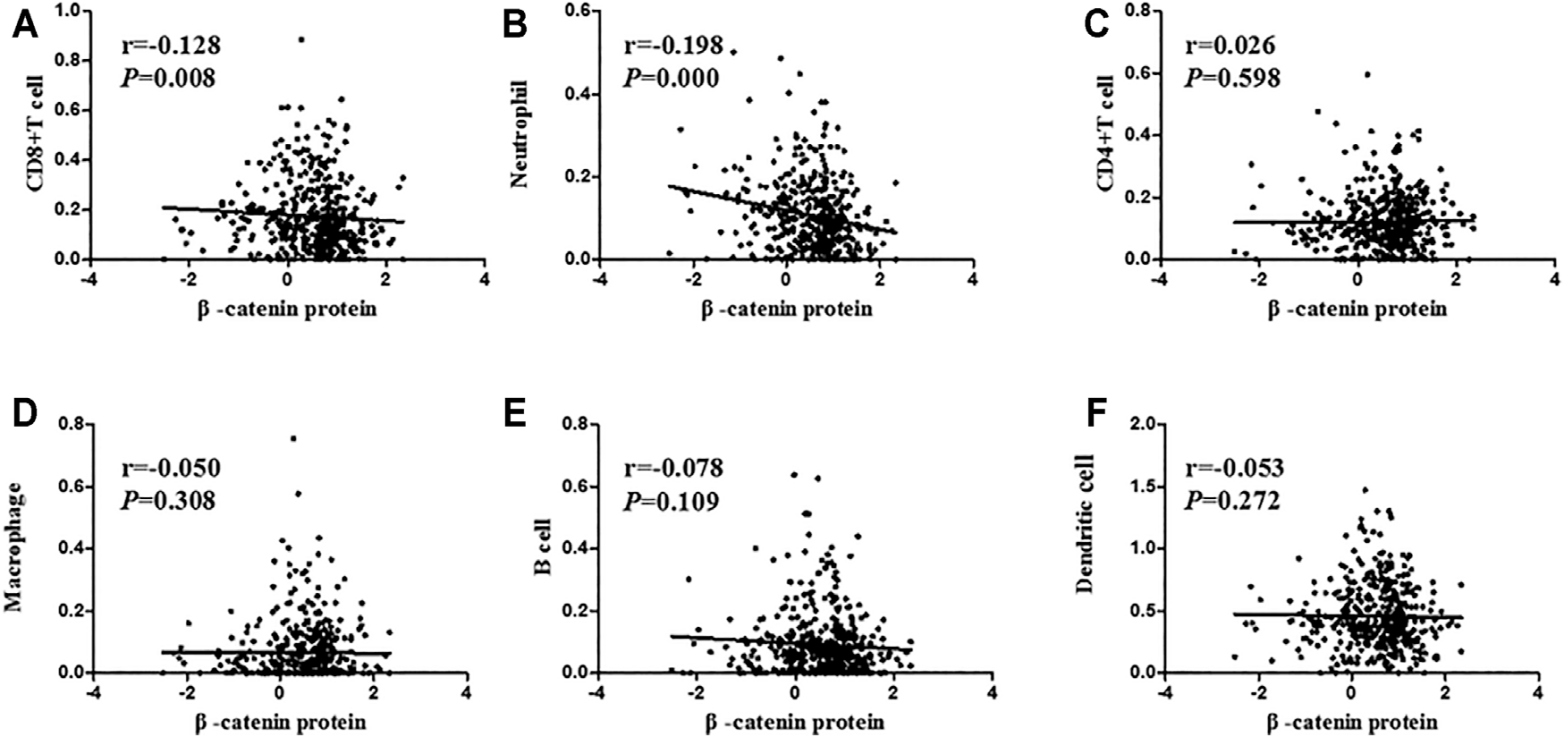
Correlation analysis between β-catenin protein and immune cell infiltration level. β-catenin protein expression had a negative link with CD8^+^ T cells (**A**; r = −0.128, *p =* 0.008) and neutrophils (**B**; r = −0.198, *p* < 0.001). β-catenin protein was not related to CD4^+^ T cells (**C**; r = 0.026, *p* = 0.598), macrophages (**D**; r = −0.050, *p* = 0.308), B cells (**E**; r = −0.078, *p* = 0.109), or dendritic cells (**F**; r = −0.053, *p* = 0.272).

Firstly, we explored the relationship between β-catenin protein and tumor-immune infiltrating cells, including CD4+ T cells, neutrophils, CD8^+^ T cells, macrophages, dendritic cells, and B cells. Expression data of β-catenin protein were downloaded from the UCSC Xena website, whose expression level was detected by reverse phase protein array (RPPA). Tumor-immune infiltrating cell data were obtained from the TIMER platform (Tumor Immune Estimation Resource). As shown in Figure 5, we found that infiltrating levels of CD8^+^ T cells (*p* = 0.008, r = −0.128) and neutrophils (*p* < 0.001, r = −0.198) in the UCSC had significant negative correlation with β-catenin protein expression level. However, there were no statistical relationships between β-catenin and CD4^+^ T cells, dendritic cells, macrophages, B cells, or β-catenin protein (All *p* > 0.05).

**FIGURE 6:**
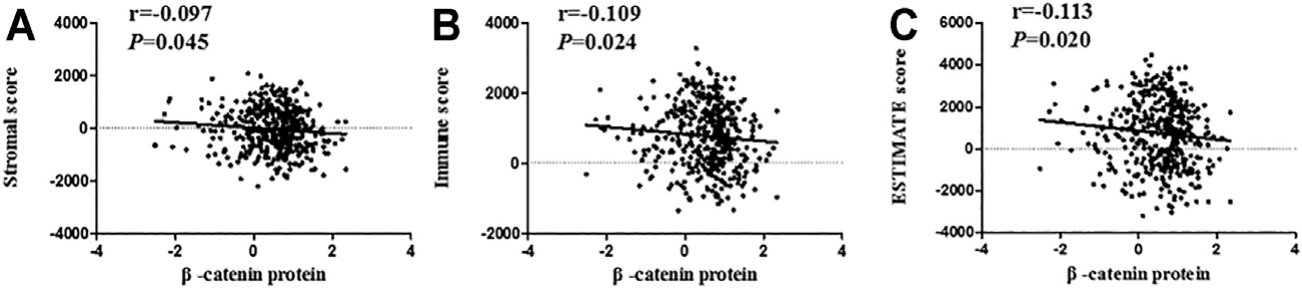
Correlation analysis between β-catenin protein and immune scores. β-catenin protein had a negative relevance with stromal score (**A**; r = −0.097, *p* = 0.045), immune score (**B**; r = −0.109, *p* = 0.024), and ESTIMATE score (**C**; r = −0.113, *p* = 0.020).

More and more evidence has shown the importance of immune and stromal cells in the immune microenvironment [24]. Stromal and immune score was a new method to calculate the infiltrating level of stromal and immune cells. In order to further study the relevance between β-catenin protein level and immune microenvironment, we also assessed the correlation between β-catenin protein and immune scores, including estimate score, immune score, and stromal score. As shown in Figure 6, we found that stromal score (*p* = 0.045, r = −0.097), immune score (*p =* 0.024, r = −0.109), and estimate score (*p =* 0.020, r = −0.113) had a significant negative correlation with β-catenin protein.

## Discussion

β-catenin is a multifunctional protein that can interact with cadherin, participate in cell adhesion, and form cytoskeleton tissue [9,10]. Most importantly, it is a critical regulating factor of the classical Wnt pathway. Wnt/β-catenin is abnormally activated in a variety of cancer patients and is closely associated with tumorigenesis, tumor growth, metastasis, and tumor immunity [14–16]. There are some reports on exploring the predictive value of β-catenin in NSCLC [25–27], but its predictive value in a Chinese cohort remains to be further explored. Therefore, this study investigated the connection between β-catenin protein and clinicopathological/prognostic factors, as well as the relevance with infiltrating tumor -immune cells and immune scores. The objective is to discover better predictive targets and to better understand the relation between the Wnt/β-catenin pathway and immune microenvironment.

In previous studies, it has been shown that higher β-catenin protein level was closely related to lymph node metastasis and low survival rate in nasopharyngeal carcinoma patients, which can be used as an indicator of prognosis [28]. Besides, β-catenin expression was reported to be associated with the resistance of NSCLC cells to gefitinib and was important for the distant metastasis of various tumors [29,30]. When the classical Wnt pathway is activated, the cytoplasmic β-catenin is transferred to the nucleus, thereby bringing about the activation of genes related to cancer development [12,13]. Hence, it may be more important to explore the value of abnormal expression of β-catenin protein. In previous literature, reduced membranous β-catenin protein defined as less than 75% of positive cells was related to distant metastasis and worse prognosis in esophageal squamous cell carcinoma patients [31]. Abnormal β-catenin expression defined as more than 10% of cells showing positive in cytoplasm/nuclei or less than 70% of cells showing positive in cytomembrane was correlated with LNM, clinical stages, and survival status of nasopharyngeal carcinoma patients [32]. Also, it has been found that hepatocellular carcinoma patients with β-catenin nuclear accumulation had poor prognosis [33]. Interestingly, β-catenin mRNA expression and its aberrant expression were both increased in LUAD and LUSC. β-catenin mRNA level was also negatively related to overall survival and post-progression survival in LUAD, but not in LUSC. We also discovered aberrant expression of β-catenin protein was associated with gender and histological type, which indicated that β-catenin protein might be used as an index of histological classification. Furthermore, we found that it was identified as a poor prognosis biomarker, which was compatible with previous reports [25–27].

The tumor microenvironment, as the living environment of tumor cells, is composed of various cytokines/chemokines, immune cells, tumor-related fibroblasts, and inflammatory cells [17,18]. ESTIMATE is a new algorithm that estimates the infiltrating grade of immune/stromal cells according to the gene expression profiles of stromal cells and immune cells [24,34]. Therefore, we used TCGA data to search the relevance between β-catenin protein and infiltrating immune cells and thereby to reveal the function of β-catenin protein in tumor immunity and tumor microenvironment. In this research, β-catenin protein was significantly negatively connected with CD8^+^ T cells and neutrophils. Meanwhile, β-catenin protein was also negatively related to ESTIMATE score, immune score, and stromal score, suggesting that β-catenin protein has a potential function in tumor immunity, which has been found in other studies [35,36]. This is the first study to simultaneously evaluate the relationship between β-catenin protein and multiple immune cells.

In summary, aberrant β-catenin protein expression was dramatically increased in NSCLC and might serve as a poor prognostic biomarker. Most importantly, β-catenin protein might play a key role in tumor immunity and the tumor microenvironment by inhibiting the infiltration of CD8^+^ T cells and neutrophils.

## Data Availability

The raw data supporting the conclusions of this article will be made available by the authors, without undue reservation.

## Supplementary Material

Supplementary Figure S1 K-M survival analysis of β-catenin protein expression in LUAD and LUSC patients. **(A)** LUAD patients with aberrant β-catenin protein expression had a shorter OS (*p* = 0.009). **(B)** β-catenin protein expression was not related to OS in LUSC ( *p* =0.349).
