# Supplementary figures and images for: Aberrant Expression of β-Catenin Correlates with Infiltrating Immune Cells and Prognosis in NSCLC
Source: Pathol Oncol Res. 2021 Oct 26;27:1609981. doi: 10.3389/pore.2021.1609981 (PMC8575687; doi:10.3389/pore.2021.1609981)

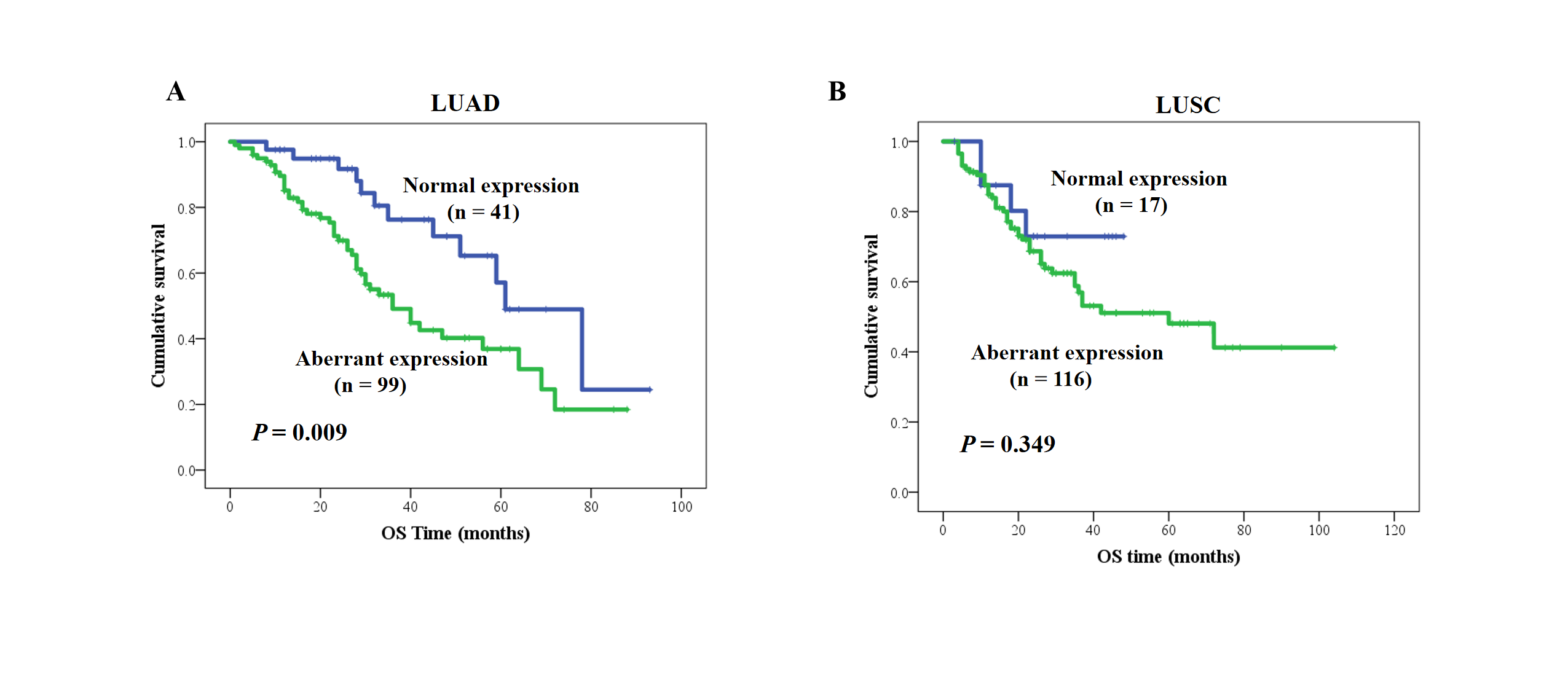

Supplement: Supplementary file 1 [file Image1.TIF]
